# Prevalence of Hypothyroidism in Patients With Erdheim-Chester Disease

**DOI:** 10.1001/jamanetworkopen.2020.19169

**Published:** 2020-10-29

**Authors:** Skand Shekhar, Ninet Sinaii, Jorge A. Irizarry-Caro, William A Gahl, Juvianee I. Estrada-Veras, Rahul Dave, Georgios Z. Papadakis, Amit Tirosh, Brent S. Abel, Joanna Klubo-Gwiezdzinska, Monica C. Skarulis, Bernadette R. Gochuico, Kevin O’Brien, Fady Hannah-Shmouni

**Affiliations:** 1 Eunice Kennedy Shriver National Institute of Child Health and Human Development, National Institutes of Health, Bethesda, Maryland; 2 Clinical Center, National Institutes of Health, Bethesda, Maryland; 3 Universidad Central del Caribe School of Medicine, Bayamón, Puerto Rico; 4 National Human Genome Research Institute, National Institutes of Health, Bethesda, Maryland; 5 Inova Fairfax-Virginia Commonwealth University College of Medicine, Falls Church; 6 Department of Radiology, Medical School, University of Crete, Heraklion, Greece; 7 Neuroendocrine Tumor Service, Sheba Medical Center, and Sackler Faculty of Medicine, Tel Aviv University, Tel Aviv, Israel; 8 Diabetes, Endocrinology, and Obesity Branch, National Institute of Diabetes and Digestive and Kidney Diseases, Bethesda, Maryland; 9 Thyroid Tumors and Functional Thyroid Disorders Section, Metabolic Disease Branch, National Institute of Diabetes and Digestive and Kidney Diseases, National Institutes of Health, Bethesda, Maryland; 10 Foundation for Research and Technology Hellas, Computational Biomedicine Laboratory, Heraklion, Greece

## Abstract

**Importance:**

Erdheim-Chester disease (ECD) is a rare non-Langerhans cell histiocytosis affecting multiple organs and commonly caused by somatic pathogenic variants in *BRAF* V600E and mitogen-activated protein kinase genes. Clinical features of ECD result from histiocytic involvement of various tissues; while endocrine involvement in ECD occurs frequently, the prevalence of central or primary hypothyroidism has not been thoroughly investigated.

**Objective:**

To assess hypothalamus-pituitary-thyroid (HPT) dysfunction in patients with ECD.

**Design, Setting, and Participants:**

This cross-sectional study included 61 patients with ECD who were enrolled in a natural history study at a tertiary care center between January 2011 and December 2018. ECD was diagnosed on the basis of clinical, genetic, and histopathological features. Data were analyzed in March 2020.

**Exposure:**

Diagnosis of ECD.

**Main Outcomes and Measures:**

Main outcome was the prevalence of thyroid dysfunction in adults with ECD compared with community estimates. Patients underwent baseline evaluation with a thyroid function test, including thyrotropin, free thyroxine (fT4), and total thyroxine (T4), and sellar imaging with magnetic resonance imaging or computed tomography scan. The association of HPT dysfunction was assessed for differences in age, sex, body mass index, *BRAF* V600E status, high sensitivity C-reactive protein level, sellar imaging, and pituitary hormonal dysfunction.

**Results:**

A total of 61 patients with ECD (46 [75%] men; mean [SD] age, 54.3 [10.9] years) were evaluated. Seventeen patients (28%) had hypothyroidism requiring levothyroxine therapy. The prevalence of both central and primary hypothyroidism were higher than community estimates (central hypothyroidism: 9.8% vs 0.1%; odds ratio, 109.0; 95% CI, 37.4-260.6; *P* < .001; primary hypothyroidism: 18.0% vs 4.7%; OR, 4.4; 95% CI, 2.1-8.7; *P* < .001). Patients with hypothyroidism (both primary and central), compared with patients with euthyroidism, had higher body mass index (median [interquartile range] 31.4 [28.3-38.3] vs 26.7 [24.4-31.9]; *P* = .004) and a higher prevalence of panhypopituitarism (7 [47%] vs 3 [7%]; *P* < .001). Among patients with hypothyroidism, those with central hypothyroidism, compared with patients with primary hypothyroidism, had a lower mean (SD) body mass index (28.3 [2.6] vs 36.3 [5.9]; *P* = .007) and higher frequencies of abnormal sellar imaging (5 [83%] vs 3 [27%]; *P* = .050) and panhypopituitarism (5 [83%] vs 3 [27%]; *P* = .050).

**Conclusions and Relevance:**

In this cohort study, a higher prevalence of central and primary hypothyroidism was identified in patients with ECD compared with the community. There should be a low threshold for testing for hypothyroidism in patients with ECD, and treatment should follow standard guidelines.

## Key Points

**Question:**

What is the prevalence of hypothalamic-pituitary-thyroid dysfunction in patients with Erdheim-Chester disease (ECD)?

**Findings:**

In this cross-sectional study of 61 patients with ECD, the prevalence of central and primary hypothyroidism was 9.8% and 18.0%, respectively, in patients with ECD, higher than the corresponding rates of 0.1% and 4.7%, respectively, in the community.

**Meaning:**

The findings of this study suggest that clinicians should consider screening for hypothyroidism in patients with ECD.

## Teaser

This cross-sectional study assesses rates of hypothalamus-pituitary-thyroid dysfunction in patients with Erdheim-Chester disease.

## Introduction

Erdheim-Chester disease (ECD) is a rare non-Langerhans cell histiocytosis affecting multiple organs including the endocrine system. While the incidence and prevalence of ECD is unknown, the medical literature contains a few hundred cases of ECD, which is characterized by male predominance and a mean age at presentation of 53 years.^1,2^ Initially described by Erdheim and Chester in 1930 as lipoid granulomatosis, the disorder was reclassified in 2016 by the World Health Organization as a neoplasm of histiocytic origin, characterized by lipid-rich foamy macrophages infiltrating various organs.^3,4,5^ Genetic perturbations in key molecular pathways, including *BRAF* V600E (OMIM 164757) and mitogen-activated protein kinase (MAPK), occur in a subset of hematopoietic cells and cause enhanced proliferation and increased survival of monocytic cells.^6,7,8^ Other genes associated with ECD include *NRAS *(OMIM 164790), *KRAS *(OMIM 190070), *ARAF *(OMIM 311010), *PIK3CA* (OMIM 171834), and *MAP2K1 *(OMIM 176872).^9,10^ The earliest manifestations of ECD include bone pain from osseous involvement (50%), followed by neurological deficits (23%) and central diabetes insipidus (22%),^1^ which is also the most misdiagnosed presenting feature of ECD.^1^ Other organ sites affected by ECD include the retroperitoneum, the cardiovascular and pulmonary systems, and the skin.^11,12,13,14,15^

Approaches to the management of ECD vary, with not all patients requiring treatment at the time of diagnosis; treatment is reserved for those with symptomatic disease, neurological involvement, or end-organ damage.^16^ Therapy options include *BRAF* V600E inhibitors, predominantly for patients with *BRAF *V600E–positive disease; pegylated interferon α for patients with *BRAF* V600E–negative disease; mitogen-activated protein kinase kinase (MEK) inhibitors for those with RAS-PI3K-AKT pathway disease–causing variants; and glucocorticoids.^16,17^

Significant endocrine organ involvement in ECD occurs primarily through the infiltration of the hypothalamic-pituitary axes.^18^ However, involvement of the hypothalamic-pituitary-thyroid (HPT) axis has not been well described in the literature.^12^ Hypothyroidism, frequently underdiagnosed in patients with ECD, may be either central (CH) or primary (PH) and may occur as the result of disease itself or as an adverse effect of certain therapies used in patients with ECD, such as therapy with interferon α. To thoroughly describe the nature, prevalence, characteristics, and factors associated with hypothyroidism in patients affected by ECD, we performed a cross-sectional study on a cohort of patients diagnosed with ECD and observed at the National Institutes of Health Clinical Center. Herein, we report on the prevalence of thyroid dysfunction and compare our estimates with general prevalence data.

## Methods

We performed a cross-sectional analysis of clinical, biochemical, and radiological characteristics of patients with ECD enrolled in an observational study. Patients diagnosed with biopsy-proven ECD were enrolled in the National Institutes of Health institutional review board–approved protocol 11 HG 0207 “Clinical and Basic Investigations into Erdheim-Chester disease” (ClinicalTrials.gov Identifier: NCT01417520) and provided written, informed consent. This report followed Strengthening the Reporting of Observational Studies in Epidemiology (STROBE) reporting guideline. Patients were enrolled between January 2011 and December 2018, and the diagnosis was established based on ECD consensus criteria^2,19^ and verified with history, physical examination, and laboratory, genetic, and imaging investigations, as previously reported.^14^ ECD biopsy samples (common sites, perinephric or retroperitoneal tissue, bone and skin) were reviewed by a certified pathologist who confirmed the diagnosis of ECD. Molecular genetic testing for *BRAF* V600E variant screening was deployed using polymerase chain reaction technique, with sequencing for exons 11 and 15 among 25 patients. Patients testing negative for *BRAF* V600E underwent testing for MAPK genes in *MAP2K1*, *PIK3CA*, *KRAS*, *NRAS*, and *ARAF* via dideoxy sequencing.

Our primary objective was to determine the prevalence of HPT dysfunction in patients with ECD and compare it with community estimates.^20,21^ The PH comparison population consisted of data from a US national normative database (National Health and Nutrition Examination Survey III)^22^ that included persons aged 12 years and older who were tested for thyrotropin, thyroxine (T4), and thyroid antibodies and diagnosed with thyroid dysfunction based on a self-reported and/or biochemical diagnosis.^20^ We derived CH prevalence estimates from published data that were mainly case reports of this rare disorder.^21^ Our secondary objective was to study the association of HPT dysfunction with age, sex, high sensitivity C-reactive protein (hs-CRP) levels, thyroid peroxidase (TPO) antibodies, abnormal results on hypothalamic-pituitary imaging, *BRAF* V600E status, and other pituitary hormonal deficits.

The laboratory workup included biochemical evaluations (serum thyrotropin, T4, and free T4 [fT4]) for all patients using immunoassay on initial presentation. We measured thyrotropin in microunits per milliliter using the Cobas 6000 immunoassay platform (Roche) (reference range, 2011-2014: 0.4-4.0 mIU/mL; 2014-2018: 0.27-4.2 mIU/mL); fT4, in nanograms per deciliter, was measured by Cobas (reference range, 2011-2014: 0.9-1.5 ng/dL; 2014-2018: 0.9-1.7 ng/dL [to convert to picomoles per liter, multiply by 12.87]), and T4, in micrograms per deciliter, measured by Cobas (reference range, 2011-2014: 4.5-12.5 μg/dL; 2014-2018: 4.5-11.7 μg/dL [to convert to picomoles per liter, multiply by 12.87]). CH was defined as a subreference thyrotropin level with a fT4 level in the low reference range in the presence of other pituitary hormone deficits or a subreference response to thyrotropin releasing hormone (TRH) stimulation test, also known as the thyrotropin-secretion test. PH was defined as thyrotropin level greater than the reference range, with low or normal fT4 level.

Overall, 7 of 17 patients (41%) requiring thyroid hormone supplementation underwent antibody testing (anti-TPO and antithyroglobulin antibodies), and 1 patient suspected of having isolated CH underwent dynamic TRH stimulation testing. For this test, TRH was administered (200 μg of synthetic TRH intravenously, with serial thyrotropin testing), and blood was drawn every 15 minutes starting 15 minutes before the injection. Thyroid ultrasonography was performed in 3 patients (5%), while 56 patients (62%) underwent sellar imaging with magnetic resonance imaging, and 5 (38%) had computed tomography scans of the brain. No individual was actively receiving interferon α, glucocorticoid, or *BRAF* inhibitor therapy at the time of enrollment. However, some had received these before enrollment: 9 of 17 patients (53%) with hypothyroidism had received interferon α treatment, of whom 3 (33%) had CH and 6 (67%) had PH. One patient (11%) had evidence of primary hypothyroidism prior to interferon α therapy.

### Statistical Analysis

Because of the use of established populations, the sample size was fixed. Data are reported as frequencies and percentages and mean and SDs or medians and interquartile ranges (IQR). The prevalence (based on frequencies and total sample sizes) of CH and PH in ECD and population estimates were compared using Fisher exact tests by constructing 2 × 2 tables, and odds ratios (ORs) and 95% (exact) CIs are reported. Continuous data were assessed for distributional assumptions and were compared between groups with HPT dysfunction (ie, hypothyroid vs euthyroid, CH vs PH) using 2-sample *t* tests or nonparametric Wilcoxon rank sum tests, as appropriate. Categorical data between groups were compared by Fisher exact tests.

While formal comparisons were focused on differences between the study cohort and the general populations, additional subgroup comparisons were added as part of descriptive statistics for exploratory purposes in the context of reporting on a rare disease. Thus, corrections for multiple comparisons were not carried out, and results should be interpreted with caution. The associations between the covariates of age, sex, disease-causing variants in *BRAF* V600E, abnormal sellar imaging, pituitary hormonal dysfunction (number of deficits and panhypopituitarism, defined as ≥3 pituitary hormonal aberrations), and the outcomes of HPT dysfunction were tested using logistic regression models. Univariable models tested individual associations to confirm results from 2-sample and categorical comparisons. However, results from only the multivariable models are reported owing to the clinical indications of the combined covariate effects. When there were missing data, the total available sample size was provided for proper interpretation of results. Statistical evidence was determined from test statistics and their corresponding indicators of uncertainty or 95% CIs along with 2-sided *P* values. Statistical significance was set at *P* ≤ .05. Data were analyzed using SAS version 9.4 (SAS Institute).

## Results

A total of 61 patients with ECD were enrolled in the protocol and included in the final analysis (46 [75%] men, mean [SD] age, 54.3 [10.9] years) (Table 1). Seventeen (28%) had hypothyroidism requiring levothyroxine (median [IQR], 125 [100-162.5] µg daily) at enrollment, with total ECD cohort median (IQR) levels within the reference range for thyrotropin (1.77 [0.95-2.74] mIU/mL; reference, 0.27-4.20 mIU/mL), fT4 (1.2 [1.1-1.3] ng/dL; reference, 0.9-1.7 ng/dL), and T4 (7.10 [5.80-8.45] µg/dL; reference, 4.5-11.7 µg/dL).

**Table 1.** Demographic and Biochemical Characteristics of Patients With Erdheim Chester Disease

Characteristic	Full cohort (N = 61)	Patients with euthyroidism (n = 44)	Patients with hypothyroidism (n = 17)^a^	Patients with central hypothyroidism (n = 6)	Patients with primary hypothyroidism (n = 11)	*P* value for euthyroid vs hypothyroid groups
Age, mean (SD), y	54.3 (10.9)	54.8 (11.1)	52.9 (10.7)	46.7 (11.7)	56.4 (8.9)	.55
Women, No. (%)	15 (25)	8 (18)	7 (41)	2 (33)	5 (45)	.10
BMI						
Median (IQR)	27.8 (24.8-32.9)	26.7 (24.4-31.9)	31.4 (28.3-38.3)	28.6 (26.3-30.4)	37.1 (30.1-38.8)	.004
Mean (SD)	29.6 (6.0)	28.17 (5.22)	33.4 (6.3)	28.3 (2.6)	36.3 (5.9)	
Thyrotropin, mIU/mL						.23
Median (IQR)	1.77 (0.95-2.74)	1.90 (1.01-2.64)	0.95 (0.20-4.19)	0.20 (0.01-0.77)	2.31 (0.27-4.86)	
Mean (SD)	1.99 (1.64)	1.93 (1.11)	2.15 (2.60)	0.36 (0.41)	3.13 (2.79)	
fT4, ng/dL, No.						
Data available, No. (%)	21 (34)	11 (25)	10 (59)	5 (83)	5 (45)	
Median (IQR)	1.20 (1.10-1.30)	1.20 (1.00-1.30)	1.20 (1.10-1.30)	1.20 (1.10-1.30)	1.20 (1.20-1.30)	.77
Mean (SD)	NA	NA	NA	1.22 (0.19)	1.22 (0.15)	
Total T4						
Data available, No. (%)	57 (93)	40 (90)	17 (100)	6 (100)	11 (100)	
Median (IQR), μg/dL	7.10 (5.80-8.45)	7.20 (5.95-8.45)	6.20 (5.70-8.45)	7.15 (5.70-9.60)	5.90 (5.60-8.45)	.44
Abnormal pituitary imaging, No. (%)	22 (36)	14 (32)	8 (47)	5 (83)	3 (27)	.37
*BRAF* V600E, positive, No./total No. (%)	31/57 (54)	23/42 (55)	8/15 (53)	4/6 (67)	4/9 (44)	>.99
Panhypopituitarism, No. (%)	11 (18)	3 (7)	8 (47)	5 (83)	3 (27)	<.001
hsCRP, median (IQR), mg/dL	1.0 (0.3-4.5)	1.2 (0.4-4.5)	0.7 (0.2-3.8)	2.8 (0.7-6.4)	0.5 (0.2-2.0)	.54
Pituitary hormonal deficits, median (IQR), No.	1.0 (0-2.0)	0 (0-1.0)	2.0 (1.0-4.0)	4.0 (3.0-4.0)	1.0 (0-3.0)	.004

Abbreviations: BMI, body mass index (calculated as weight in kilograms divided by height in meters squared); fT4, free thyroxine; hsCRP, high sensitivity C-reactive protein; IQR, interquartile range; T4, thyroxine.

SI conversion factors: To convert fT4 to picomoles per liter, multiply by 12.87; hcCRP to milligrams per liter, multiply by 10; T4 to nanomoles per liter, multiply by 12.87.

^a^ Median (IQR) time to hypothyroidism diagnosis was 2.0 (1.0-6.5) years.

Body mass index (BMI; calculated as weight in kilograms divided by height in meters squared) was higher in patients with hypothyroidism compared with those with euthyroidism (median [IQR], 31.4 [28.3-38.3] vs 26.7 [24.4-31.9]; *P* = .004). The prevalence of abnormal pituitary imaging (8 of 17 patients [47%] vs 14 of 44 [32%]; difference, 15%; 95% CI, 12%-43%; *P* = .37) was not different, although panhypopituitarism occurred more frequently in the hypothyroid group compared with the euthyroid group (8 [47%] vs 3 [7%]; difference, 40%; 95% CI, 15%-65%; *P* < .001) (Table 1).

Of 17 patients with hypothyroidism, 6 (35%) had CH (mean [SD] thyrotropin level, 0.36 [0.41] mIU/mL; mean [SD] fT4 level, 1.22 [0.19] ng/dL; median [IQR] T4 level, 7.15 [5.70-9.60] μg/dL) (Table 1), while 11 (65%) had PH. The prevalence of PH in ECD was 4 times higher than community estimates^20^ (18.0% vs 4.7%; OR, 4.4; 95% CI, 2.1-8.7; *P* < .001). Given its rare occurrence, CH was 109 times more prevalent in patients with ECD compared with population estimates^21^ (9.8% vs 0.1%; OR, 109.0; 95% CI, 37.4-260.6; *P* < .001). Moreover, among those with CH, diabetes insipidus and central hypogonadism were the most frequent concurrent pituitary deficiencies, occurring in 4 of 6 patients (67%). One patient with CH (17%) had coexisting diabetes insipidus and central adrenal insufficiency, and another patient (17%) had no pituitary hormone deficit other than CH.

CH and PH were similarly distributed between sexes (Table 1). Mean (SD) BMI was higher in patients with PH compared with those with CH (36.3 [5.9] vs 28.3 [2.6]; *P* = .007) (Table 1). No patient presented with myxedema coma or thyrotoxicosis. Of 11 patients with suspected PH, 7 (63%) underwent TPO testing; 4 (57%) had positive results, and 3 (43%) had negative results for TPO antibodies. Those with PH who had elevated antithyroid antibodies had the following values: 355 U/mL, greater than 1000 U/mL, 52 U/mL, and 47 U/mL, with the upper reference limit being 35 U/mL. Three (43%) patients had normal thyroid ultrasonography, all of whom had CH. Six patients had 1 or more historical factors suggestive of CH, including a history of thyroid hormone supplementation, coexisting hormonal deficiencies (Table 1), and biochemical test results consistent with a CH etiology.

### Factors Associated With Hypothyroidism in Patients With ECD

Based on multivariable logistic regression analysis limited by sample size but nonetheless informative for clinical care, only female sex increased the odds of hypothyroidism (OR, 19.6; 95% CI, 3.0-129.4; *P* = .002); age (OR, 1.1; 95% CI, 1.0-1.2), pathogenic variants in *BRAF* V600E (OR, 0.7; 95% CI, 0.1-3.5), abnormal sellar imaging (OR, 0.7; 95% CI, 0.1-4.3), and pituitary hormonal dysfunction (OR, 2.2; 95% CI, 0.7-6.6) had no influence. No associations from these multivariable effects were observed for CH vs PH.

**Figure 1.**
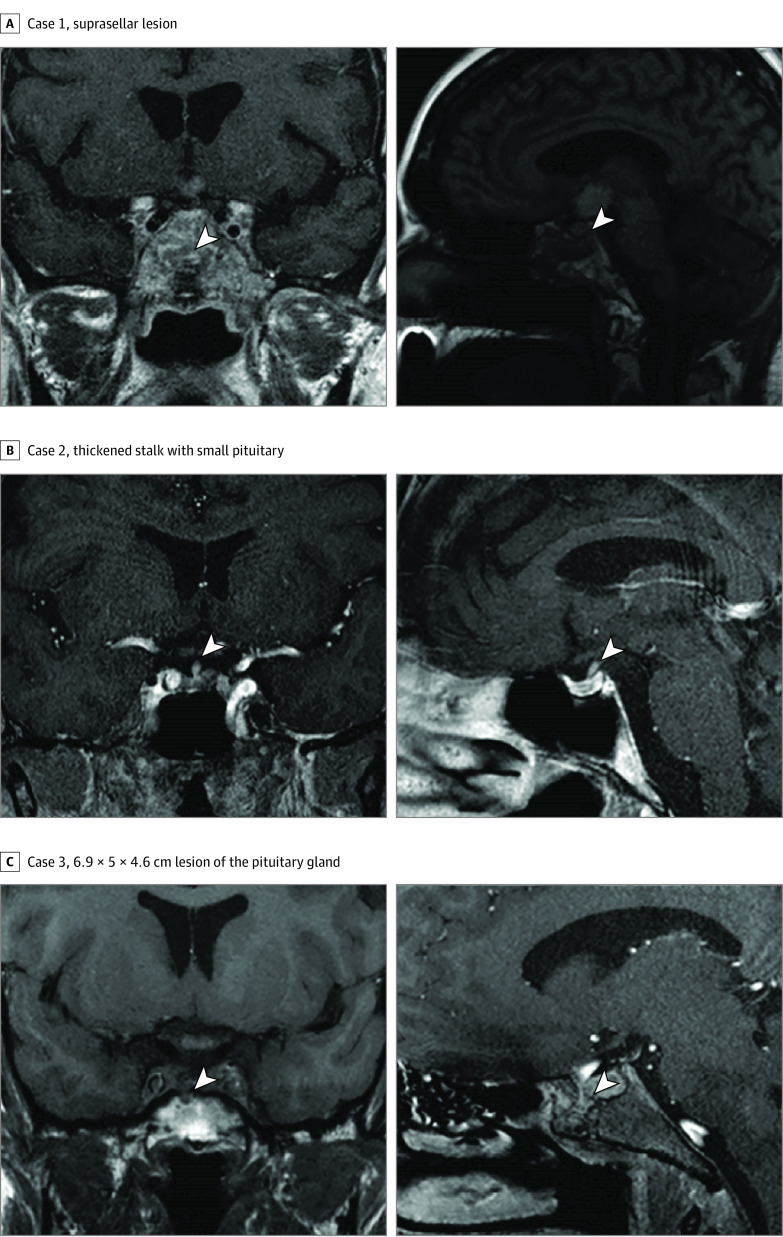
T1-Weighted Magnetic Resonance Imaging Scans of 3 Patients With Central Hypothyroidism

While sample sizes were small in albeit rare conditions of ECD and CH, no difference in median (IQR) hsCRP was observed in CH compared with PH (2.8 [0.7-6.4] mg/dL vs 0.5 [0.2-2.0] mg/dL [to convert to milligrams per liter, multiply by 10]; *P* = .12), but mean (SD) thyrotropin values were higher in patients with PH than those with CH (3.13 [2.79] mIU/mL vs 0.36 [0.41] mIU/mL; *P* = .008); fT4, T4, and levothyroxine dose levels were similar between patients with CH and PH. The prevalence of abnormal sellar imaging (5 [83%] vs 3 [27%]; difference, 56%; 95% CI, 16%-96%; *P* = .050) and panhypopituitarism (5 [83%] vs 3 [27%]; difference, 56%; 95% CI 16%-96%; *P* = .050) may be higher in patients with CH (Figure 1; eFigure in the Supplement). The total number of pituitary hormone deficits appeared to be higher in patients with CH compared with those with PH (median [IQR], 4 [3-4] vs 1 [0-3]; *P* = .02) (Table 1). We could not determine an association between anti-TPO antibodies and CH vs PH due to the small number of patients with these measurements.

### Illustrative Case of ECD With Isolated Central Hypothyroidism

A White woman in her early sixties with a pathogenic variant in *BRAF* V600E underwent evaluation for CH. She had cerebellar dysfunction, retroperitoneal fibrosis, and osteosclerosis and was asymptomatic for thyroid dysfunction. Her baseline anterior pituitary hormones were within the reference range, with the exception of a low thyrotropin level (Table 2). Her thyrotropin level rose from 0.33 mIU/mL at baseline to a subreference value of 2.90 mIU/mL at 90 minutes (reference, change in thyrotropin of >7 mIU/mL) during the TRH stimulation test, confirming isolated CH. Magnetic resonance images of the pituitary gland showed a small hypoenhancing lesion in the posterior aspect of the pituitary gland (eFigure 1, case 4, in the Supplement).

**Table 2.** Anterior Pituitary Hormones Fasting Levels and Normative Reference Ranges in Index Case With Isolated Central Hypothyroidism

Test	Value	Reference range
Insulin-like growth factor 1, ng/mL	140	143-859
Growth hormone, ng/mL	0.09	0-8
Follicle-stimulating hormone, mIU/mL	49.8	22-153
Luteinizing hormone, mIU/mL	26.3	11-40
Thyrotropin, mIU/mL		0.27-4.2
Measurement 1	0.16	
Measurement 2	0.20	
Measurement 3	0.38	
Adrenocorticotropic hormone, pg/mL	35.2	0-46
Cortisol, µg/dL	14.9	5-25
Prolactin, ng/mL	19.5	2-25
Change in thyrotropin after TRH test, mIU/mL	2.57	>7

Abbreviations: TRH, thyroropin-releasing hormone.

SI conversion factors: To convert adrenocorticotropic hormone to picomoles per liter, multiply by 0.22; cortisol to nanomoles per liter, multiply by 0.036; follicle-stimulating hormone and luteinzing hormone to units per liter, multiply by 1.0; growth hormone to micrograms per liter, multiply by 1.0; insulinlike growth factor to nanomoles per liter, multiply by 0.131; prolactin to micrograms per liter, multiply by 1.0.

## Discussion

Our large cross-sectional study of ECD patients found a higher prevalence of both CH and PH compared with community estimates. Hypothyroidism was associated with higher frequencies of abnormal pituitary imaging, female sex, higher BMI, and panhypopituitarism. In the group with hypothyroidism, CH was more likely to occur in patients who had a lower BMI, lower thyrotropin level, abnormal pituitary imaging, and panhypopituitarism (Table 2 and Table 3).^12,23,24,25,26^ We also identified a case of isolated CH confirmed with a TRH stimulation test in the absence of other hypothalamic-pituitary hormonal axis dysfunction.

**Table 3.** Illustrative Cases of Previously Reported Central Hypothyroidism in Patients with Erdheim-Chester Disease

Source	Age, y	Sex	Imaging	Hormone axis deficits
Kovacs et al,^23^ 2004	35	Female	Lesions in pituitary stalk	ACTH, thyrotropin, LH, FSH, DI
Abla et al,^24^ 2010	26	Female	Suprasellar enhancement within the infundibulum, optic apparatus, and inferior hypothalamus	DI, LH, FSH, thyrotropin, ACTH, IGF-1
Pineles et al,^25^ 2011	32	Female	Neuroimaging revealed an infiltrative process within the pituitary gland	Panhypopituitarism
Manaka et al,^26^ 2014	16	Male	Thickened pituitary stalk and enlarged anterior lobe with delayed enhancement	Gonadotropin, testosterone, ACTH, cortisol, GH, IGF-1
Courtillot C et al,^12^ 2016	NR	NR	NR	NR

Abbreviations: ACTH, adrenocorticotropic hormone; DI, diabetes insipidus; ECD, Erdheim-Chester disease; FSH, follicle-stimulating hormone; GH, growth hormone; IGF-1 insulinlike growth factor 1; LH, luteinizing hormone; NR, not reported.

The presence of thyroid involvement in ECD was first reported in the form of lipogranulomatosis of the thyroid.^27^ Thyroid dysfunction through direct infiltration of the glandular parenchyma has been reported^28^; in our cohort, thyroid tissue was not examined because no patient underwent thyroid biopsy or surgery. We identified a 4-fold higher prevalence of hypothyroidism in ECD compared with population estimates, suggesting a direct or indirect association of the disease process with thyroid function.^20^ Notably, the proportion of women in the National Health and Nutrition Examination Survey III study (52.8%) was higher than in our ECD population (25%).

The prevalence of CH was 9.8% in our ECD cohort, compared with approximately 0.1% in the general population, which is consistent with previous estimates.^12,21^ Elevated hsCRP levels have been associated with infiltrative hypophysitis^29^ and indicate a higher inflammatory burden. While this was not observed, a substantially higher prevalence of hypothyroidism seen in our cohort of patients with ECD, with both TPO positive and negative forms, raises the possibility that both direct infiltration of the thyroid as well as antibody-mediated dysfunction could play important roles in the pathophysiology of hypothyroidism in ECD.

Interferon α as well as other chemotherapeutic agents used to treat ECD may contribute to the spectrum of thyroid dysfunction.^30^ In this report, no enrolled patients were actively treated with interferon α therapy at the time of enrollment; however, 9 of 17 patients with hypothyroidism (53%) had a history of exposure to interferon α. A causal relationship between previous medication exposure and hypothyroidism could not be determined in our analysis.

**Figure 2.**
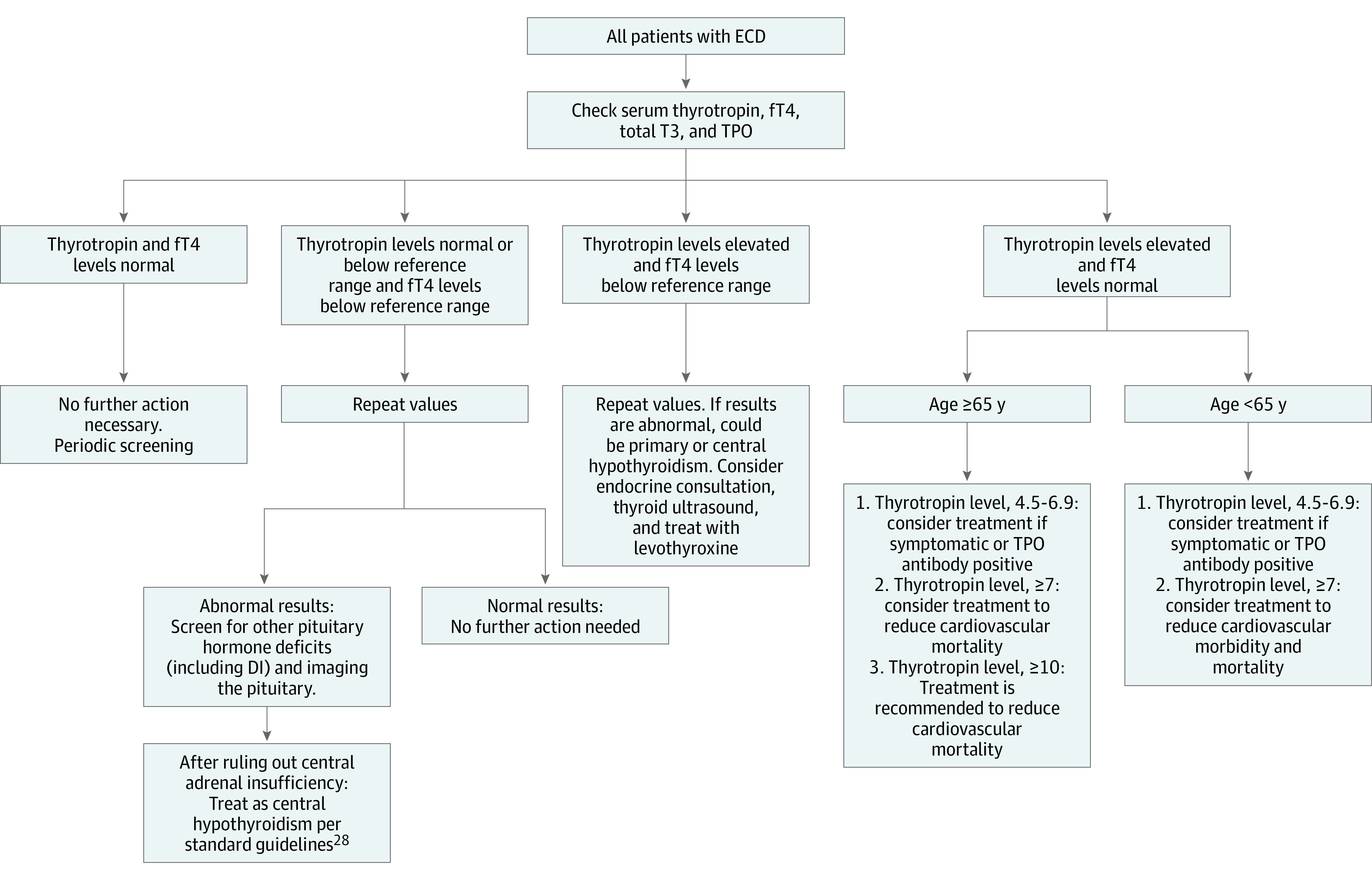
Algorithm to Screen for Hypothyroidism in Patients With Erdheim-Chester Disease (ECD) DI indicates diabetes insipidus; fT4, free thyroxine; T3, triiodothyronine; TPO, thyroid peroxidase.

A review of the literature revealed a total of 4 previous cases of overt CH in ECD (Table 3), all in the setting of panhypopituitarism.^12,23,24,25,26^ Three patients (75%) were women, with an age range of 16 to 35 years at the time of diagnosis, and all had abnormal pituitary imaging. Our case of isolated CH is unique in that it was not associated with other pituitary dysfunctions and the TRH stimulation test, which is the criterion standard despite being rarely available for ascertaining CH, confirmed the finding. Clinicians should be aware of HPT dysfunction in ECD, and we recommend that all patients diagnosed with ECD be screened at baseline for hypothyroidism with measurements of thyrotropin, circulating thyroid hormones (fT4 and triiodothyronine [T3]), and anti-TPO antibodies (Figure 2). Thyrotropin alone should not be used to diagnose or observe patients with CH, given that thyrotropin levels can be low, in the reference range, or high, and the secretion of thyrotropin with low bioactivity may account for the lack of correlation between immunoreactive and other biochemical thyroid parameters. Thus, and as shown in our illustrative case, an attenuated increment in thyrotropin after stimulation by TRH is required for diagnosis. Furthermore, patients who are diagnosed with CH and started on thyroid hormone replacement should be monitored with circulating thyroid hormones to determine adequacy of the replacement dose, considering that thyrotropin is not useful to determine euthyroid status in such individuals. Reliance on circulating thyroid hormones for diagnosis is thus important. Subclinical hypothyroidism (in certain situations), defined as elevated thyrotropin levels with fT4 levels in the reference range, and CH warrant treatment to reduce cardiovascular morbidity and mortality.^31^ We propose an algorithm for screening and management of HPT dysfunction in patients with ECD in accordance with standard guidelines^32^ (Figure 2).

### Limitations

Our study has several limitations. First, this was a cross-sectional analysis of patients who were enrolled for ECD evaluation; therefore, a causal relationship between ECD and thyroid dysfunction cannot be confirmed based on the study design. Second, the protocol did not include various endocrine evaluations including thyroid ultrasonography, fT4, total T3, and TPO on all patients given its inherent design. Third, we were not able to assess the associations of medications, such as interferon α or glucocorticoids, with the endocrine system, including the HPT axis. Fourth, we were unable to calculate incidence of hypothyroidism during follow-up or determine time to development of hypothyroidism because this was a cross-sectional study. However, this study distinguished the rare occurrence of CH and reported on details related to HPT dysfunction. In this context of small sample sizes and multiple comparisons, it is important to interpret results with caution.

## Conclusions

This cross-sectional study reported a high prevalence of CH and PH in patients with ECD, suggesting the need to screen all patients with ECD for hypothyroidism. Furthermore, abnormal pituitary imaging, female sex, BMI in the obese range, and panhypopituitarism were found to be associated with hypothyroidism. Further studies are needed to examine the underlying pathophysiology of hypothyroidism in ECD.

## Notes

Supplement. **eFigure. **T1-Weighted MRI Brain Scans of 3 Patients (Cases 4, 5, and 6) With Central Hypothyroidism
