# Supplementary material for: Prevalence of Hypothyroidism in Patients With Erdheim-Chester Disease
Source: JAMA Netw Open. 2020 Oct 29;3(10):e2019169. doi: 10.1001/jamanetworkopen.2020.19169 (PMC7596581; doi:10.1001/jamanetworkopen.2020.19169)
Supplement: Supplement. — eFigure. T1-Weighted MRI Brain Scans of 3 Patients (Cases 4, 5, and 6) With Central Hypothyroidism [file jamanetwopen-e2019169-s001.pdf]

Supplemental Online Content

Shekhar S, Sinaii N, Irizarry-Caro JA, et al. Prevalence of hypothyroidism in patients with Erdheim-Chester disease. *JAMA Netw Open*. 2020;3(10):e2019169. doi:10.1001/jamanetworkopen.2020.19169

eFigure. T1-Weighted MRI Brain Scans of 3 Patients (Cases 4, 5, and 6) With Central Hypothyroidism

This supplemental material has been provided by the authors to give readers additional information about their work.

© 2020 Shekhar S et al. *JAMA Network Open.*

eFigure. T1-Weighted MRI Brain Scans of 3 Patients (Cases 4, 5, and 6) With Central Hypothyroidism

Each horizontal panel represents one patient's pituitary imaging.

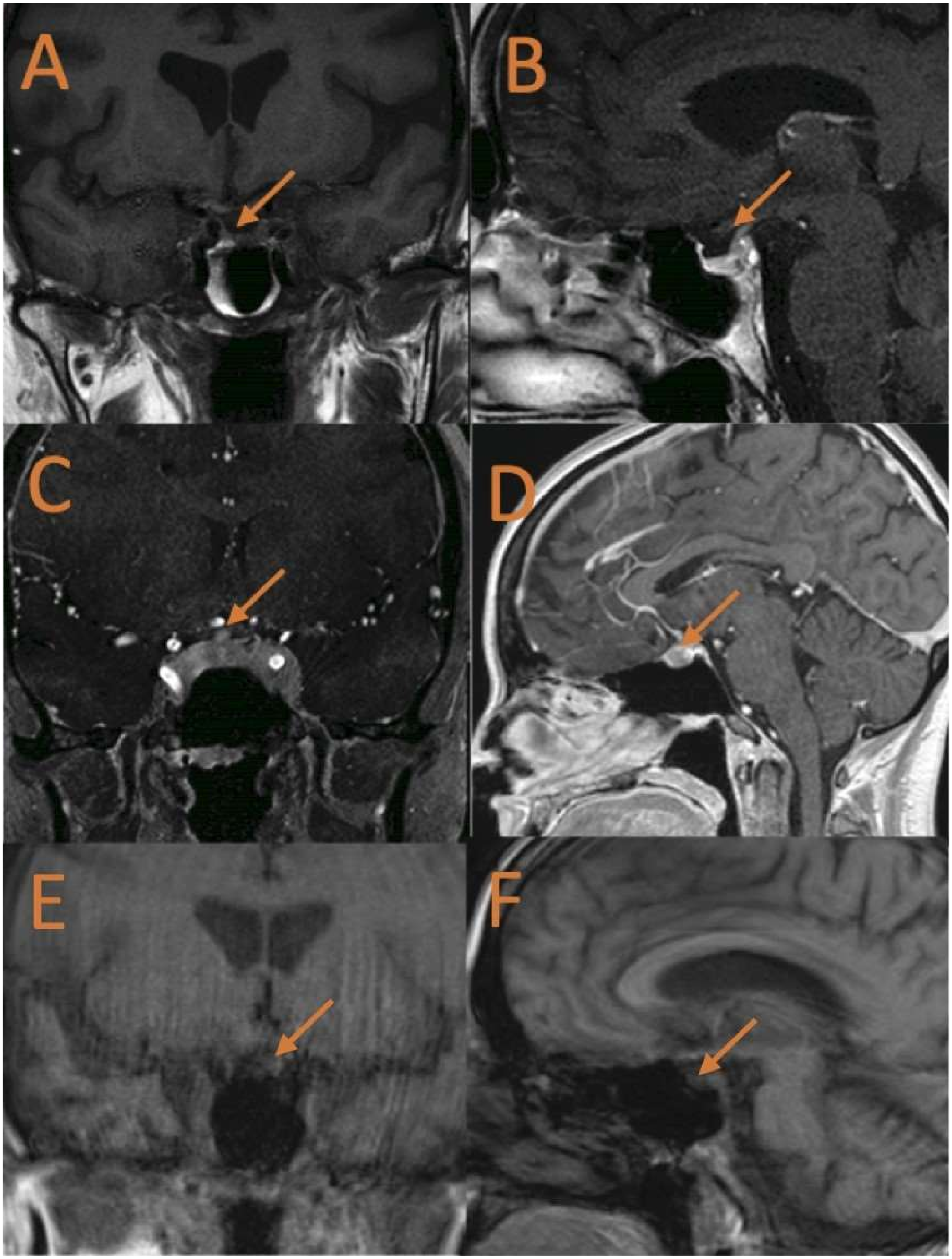

© 2020 Shekhar S et al. *JAMA Network Open.*
